# Emerging Bioactive Agent Delivery-Based Regenerative Therapies for Lower Genitourinary Tissues

**DOI:** 10.3390/pharmaceutics14081718

**Published:** 2022-08-17

**Authors:** Lin-Cui Da, Yan Sun, Yun-Hong Lin, Su-Zhu Chen, Gang-Xin Chen, Bei-Hong Zheng, Sheng-Rong Du

**Affiliations:** 1 Center of Reproductive Medicine, Fujian Maternity and Child Health Hospital College of Clinical Medicine for Obstetrics & Gynecology and Pediatrics, Fujian Medical University, Fuzhou 350001, China; 2 The Key Laboratory of Innate Immune Biology of Fujian Province, Biomedical Research Center of South China, College of Life Sciences, Fujian Normal University, Fuzhou 350117, China

**Keywords:** controlled release, regeneration medicine, urinary system injury, reproductive system injury

## Abstract

Injury to lower genitourinary (GU) tissues, which may result in either infertility and/or organ dysfunctions, threatens the overall health of humans. Bioactive agent-based regenerative therapy is a promising therapeutic method. However, strategies for spatiotemporal delivery of bioactive agents with optimal stability, activity, and tunable delivery for effective sustained disease management are still in need and present challenges. In this review, we present the advancements of the pivotal components in delivery systems, including biomedical innovations, system fabrication methods, and loading strategies, which may improve the performance of delivery systems for better regenerative effects. We also review the most recent developments in the application of these technologies, and the potential for delivery-based regenerative therapies to treat lower GU injuries. Recent progress suggests that the use of advanced strategies have not only made it possible to develop better and more diverse functionalities, but also more precise, and smarter bioactive agent delivery systems for regenerative therapy. Their application in lower GU injury treatment has achieved certain effects in both patients with lower genitourinary injuries and/or in model animals. The continuous evolution of biomaterials and therapeutic agents, advances in three-dimensional printing, as well as emerging techniques all show a promising future for the treatment of lower GU-related disorders and dysfunctions.

## 1. Introduction

The lower genitourinary (GU) system refers to the urinary and reproductive organs apart from the kidney and ureter, such as the urethra, bladder, ovaries, uterus, vagina, scrotum, penis, and testes [1]. Lower GU trauma, usually caused by disease, accident, or iatrogenesis, has long been an important medical problem because the affected tissues generally fail to regenerate after injury, resulting in serious urinary, sexual, reproductive, and psychological consequences [2,3,4]. Therefore, improved strategies are urgently needed for the structural and functional reconstruction of damaged lower GU tissues.

Regenerative medicine, a multidisciplinary field that seeks to efficiently repair and regenerate injured tissues after trauma, has emerged as an attractive option for the treatment of lower GU damage [5,6,7]. Further, the use of bioactive agents (e.g., drugs, growth factors, cytokines, hormones, inhibitors, genes, and even living cells) to modulate cellular behavior and treat tissues may help improve current regenerative medicine approaches [8,9,10]. However, the direct application of bioactive agents in regenerative medicine is limited by their lack of stability, solubility, and ease of migration from the application site, making them ineffective for sustained treatment [5,11]. A delivery system refers to a device or formulation that enables spatiotemporal controlled release of active substances with adequate dosage and correct form at the target site [12,13,14]. Delivery systems aim to enhance the bioavailability of active substances, extend the duration of pharmacological action, increase treatment efficacy, and reduce adverse effects, thereby, acting as an important driving force for regenerative therapy [13].

However, regarding the delivery of active compounds, there is still a significant gap for improvement in loading efficiency, stability, therapeutic activity, and their spatiotemporal controlled release profile. Therefore, various exploration steps have been taken to develop sophisticated bioactive agent delivery systems focused on the types of biomaterials, delivery systems, and loading strategies. For instance, to endow biomaterials with additional characteristics that are beneficial to regenerative therapies, different composite materials, crosslinking methods, and chemical modifications have been developed [15,16,17,18]. Furthermore, fabrication methods which may affect drug encapsulation, release, and biological efficacy, such as cell exosome production, three-dimensional (3D) printing, electrospinning, and microfluidics, have been used to produce diverse forms of delivery systems for the sequential delivery of therapeutic substances [19,20,21,22,23]. In addition, many types of loading strategies, such as adding stabilizing excipients, covalent immobilization, in vitro loading, and structure optimization have been utilized to enhance bioavailability and prevent burst release [24,25,26,27].

Considering the extensive advanced delivery strategies currently used in regenerative therapy, the aim of this review is to provide a comprehensive summary of the key aspects of bioactive agent-based delivery system design and established techniques, and further discuss their applications and potential for treating lower GU injuries. We believe that this review will promote the interdisciplinary communication between pharmaceutics, regenerative medicine, and GU medicine, and inspire new innovations in engineered lower GU tissue research.

## 2. Classification of Biomaterials Used in Delivery Systems

A delivery system generally utilizes biomaterials as carriers to embed, deliver, and release bioactive substances at the desired site under controlled conditions [28,29]. Among them, inorganic and organic biomaterials are popular in delivery system-based regenerative medicine.

### 2.1. Inorganic Biomaterials

**Figure 1:**
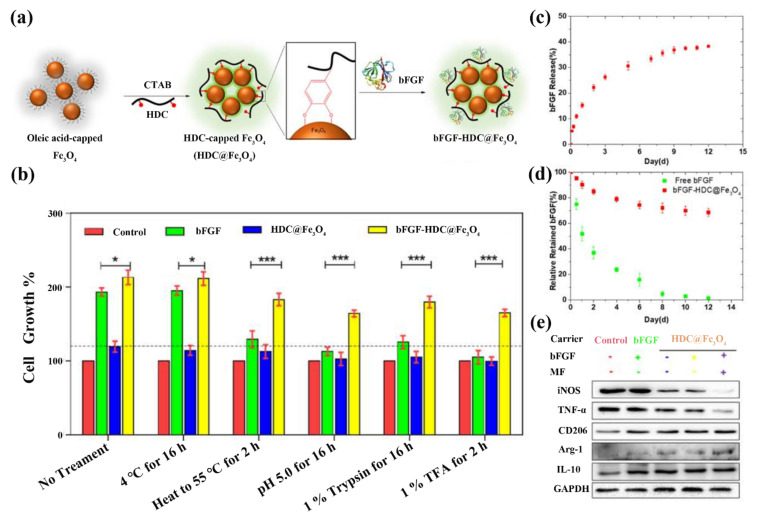
(**a**) Diagrammatic sketch of bFGF-HDC@Fe_3_O_4_ fabrication; (**b**) cell growth rate of NIH 3T3 cultures in groups of free bFGF, HDC@Fe_3_O_4_, and bFGF-HDC@Fe_3_O_4_ exposed to various conditions, for statistical significance, * *p* < 0.05 and *** *p* < 0.001; (**c**) bFGF release behavior of bFGF-HDC@Fe_3_O_4_; (**d**) bFGF bioactivity evaluation of bFGF-HDC@Fe_3_O_4_ (red) and free bFGF (green); (**e**) expression analysis of proinflammatory macrophage-associated proteins (iNOS and TNF-α) and anti-inflammatory phenotype macrophage-associated proteins (CD206, Arg-1, and IL-10) through Western blot. CTAB, hexadecyl trimethyl ammonium bromide; HDC, dopamine heparin conjugate; HDC@Fe_3_O_4_, heparin dopamine conjugate-coated Fe_3_O_4_ nanoparticles; bFGF, basic fibroblast growth factor; TFA, trifluoroacetic acid. Adapted with permission from [25], published by the American Chemical Society, 2021.

Inorganic biomaterials, including but not limited to metals, metallic oxides, and glasses, have been explored to fabricate delivery systems for soft tissue therapeutic applications. Metals and metallic oxides are considered to be potential carriers because of their well-defined structures and ease of chemical functionalization [30]. In addition to their carrier function, some metals or metallic oxides, such as gold, silver, iron oxide, zinc oxide, and cerium oxide, possess interesting features such as antibacterial activity, antioxidant properties, and the capacity to magnetically drive macrophage polarization [31,32]. Iron oxide nanoparticles are an excellent example of an inorganic biomaterial that has been intensively studied for use in regenerative therapy. Wu et al. fabricated basic fibroblast growth factor (bFGF)-loaded heparin dopamine conjugate-coated Fe_3_O_4_ nanoparticles (bFGF-HDC@Fe_3_O_4_) through surface immobilization (Figure 1a) [25]. The stability and bioactivity of bFGF was tested by evaluating the effect on NIH 3T3 cell viability after being reacted with bFGF-HDC@Fe_3_O_4_ nanoparticles at various conditions that may be encountered during preparation, storage, or application (e.g., 4 °C, 55 °C, pH 5.0, 1% trypsin, and 1% trifluoroacetic acid) [25]. The cell growth rate was much higher in the bFGF-HDC@Fe_3_O_4_ group than in the free bFGF group, particularly under harsh conditions, demonstrating that HDC@Fe_3_O_4_ was capable of effectively maintaining the stability of bFGF (Figure 1b) [25]. These nanoparticles also demonstrated good stability and controlled release, gradually releasing 40% of the bFGF and retaining 70% protein activity over 12 days (Figure 1c,d) [25]. Moreover, with the help of an external magnetic field, bFGF-HDC@Fe_3_O_4_ nanoparticles were efficiently distributed to the mitochondria of macrophages, thus, promoting anti-inflammatory phenotype macrophage polarization to accelerate tissue regeneration (Figure 1e) [25]. In 2020, Khosravi et al. developed curcumin-loaded superparamagnetic iron oxide nanoparticles (SPIONs) to treat testes damage caused by heat stress [33]. In another study, ferucarbotran, a commercial agent composed of SPIONs, was used to deliver bone marrow mesenchymal stem cells (MSCs) for the repairment of resected bladder tissue [34]. Using this method, the MSCs acquired magnetic characteristics allowing their accumulation in damaged areas under the direction of an external magnetic field, effectively enhancing tissue regeneration in a minimally invasive approach [34].

Mesoporous glass (e.g., borates, silicates, and phosphates) nanoparticles have been studied as carriers for regenerative medicine owing to their remarkable physicochemical properties, including ease of synthesis and functionalization, low mass density, controllable nanoparticle size, tunable microstructure, high specific surface area, and cytocompatibility [35]. In 2020, Hamam et al. developed curcumin-loaded mesoporous silica particles for tissue regeneration [36]. In another example, ultrasmall ceria nanocrystals with controlled reactive oxygen species (ROS) scavenging capability were loaded on uniform mesoporous silica nanoparticles to alleviate oxidative damage at the injury site [37]. These ceria-loaded nanoparticles induced regenerative healing effects, indicating their great potential for wound repair applications in which ROS-scavenging activity is beneficial [37]. In addition, Wang et al. developed poly(amidoamine) dendrimers modified mesoporous silica nanoparticles with controlled drug release properties for bladder cancer therapy [38]. This delivery system showed excellent mucoadhesive capabilities on bladder wall, which could provide enlightenment for the development of bioactive agent-based delivery systems for bladder regenerative treatment.

### 2.2. Organic Biomaterials

Organic biomaterials can be divided into synthetic polymeric and bioderived materials, depending on their source. Polymeric materials that are accurately synthesized through reproducible industrial processes have been widely utilized in regenerative medicine applications because of their tunable physicochemical properties [39,40]. In addition, these materials are popular for the sustained or controlled release of encapsulated active substances. Thus, polymeric materials, such as polylactide-co-glycolide (PLGA), polyethylene glycol (PEG), polyvinyl alcohol (PVA), and poly (ester amide) (PEA) are suitable candidates for carriers of bioactive agents in tissue regeneration. The possible release mechanisms of bioactive agents from synthetic polymers include time and condition-dependent surface erosion, desorption, and swelling and diffusion [14].

PLGA, which possesses a tunable degradation rate and induces minimal systemic cytotoxicity, is an attractive FDA-approved polyester in applications that require long-term delivery of therapeutic substances. Ciardulli et al. encapsulated human growth differentiation factor 5 in a PLGA nanocarrier for controlled delivery, thus, promoting tissue regeneration events [41]. PEG is another polyester that has received significant attention for its tunable geometry and hydrophilicity. Recently, Sok et al. developed a PEG-based hydrogel that showed promising results for delivery of aspirin-triggered resolvin D1 and recombinant human interleukin 10, resulting in recruitment of immune cells, their polarization towards pro-regenerative phenotypes, and subsequent healing of trauma wounds [42]. Liang et al. fabricated PEG—poly (ε-caprolactone-co-lactide)-based thermosensitive delivery system to deliver adipose stem cell-derived exosomes under sustained manner in corpus cavernous, and finally ended with erectile function restored [22].

**Figure 2:**
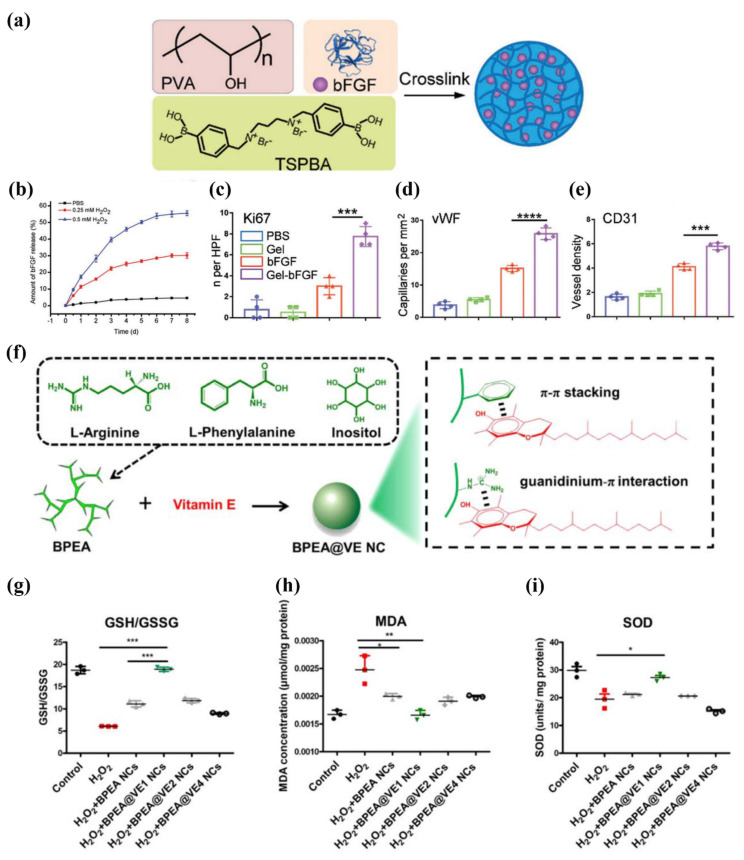
(**a**) Diagrammatic sketch of the preparation of bFGF loaded with PVA-TSPBA; (**b**) bFGF release behavior of bFGF loaded with PVA-TSPBA at various ROS concentrations. Quantitative data corresponding to Ki67 staining (**c**), vWF staining (**d**), and CD31 staining (**e**) (for statistical significance, *** *p* < 0.005 and **** *p* < 0.0001 (**c**–**e**)); (**f**) schematic illustration of BPEA@VE NC; (**g**) GSH/GSSG values in H_2_O_2_ and in BPEA@ VE NC-treated cells (for statistical significance, *** *p* < 0.001); (**h**) MDA concentration in H_2_O_2_ and BPEA@ VE NC-treated cells (for statistical significance, * *p* < 0.05 and ** *p* < 0.01); (**i**) SOD concentration in H_2_O_2_ and BPEA@ VE NC-treated cells (for statistical significance, * *p* < 0.05). bFGF, basic fibroblast growth factor; PVA, poly (vinyl alcohol); TSPBA, N1-(4-boronobenzyl)-N3-(4-boronophenyl)-N1, N1, N3, N3-tetramethylpropane-1, 3-diaminium; BPEA@VE NCs, branched poly (ester amide) nanocapsules loaded with vitamin E; GSH/GSSG, reduced glutathione/oxidized glutathione disulfide; MDA, malondialdehyde; SOD, superoxide dismutase. Adapted with permission from [43,44], published by Wiley, 2021 and American Chemical Society, 2021.

PVA is a food and drug administration (FDA) approved polyol that can acquire ROS-responsive capacity after reacting with benzoboric acid [43]. Li et al. developed a hydrogel composed of PVA and benzoboric acid to deliver bFGF, demonstrating promising results for the repair of tissues with high ROS microenvironments (Figure 2a–e) [43]. PEA, a cationic polymer consisting of ester and amino groups synthesized from natural active biomolecules, is nontoxic and possesses excellent biodegradability, biocompatibility, and mechanical properties [44]. Yuan et al. recently synthesized PEA using L-arginine, L-phenylalanine, and inositol as raw materials (Figure 2f) [44]. Vitamin E encapsulated in PEA showed excellent antioxidative and anti-inflammatory properties in tissue engineering applications (Figure 2g–i) [44]. In 2020, PEA-plasmid polyplex-based delivery systems were used successfully to deliver exogenous deoxyribonucleic acid to the vagina/cervix without diffusing to nearby organs, which showed it immense application potential in vagina/cervix regenerative therapies [45].

Bioderived materials are defined as macromolecules extracted from microorganisms, animals, or plants [46,47,48]. Advantages such as high availability, biocompatibility, and bioactivity have facilitated the use of bioderived materials as delivery systems for therapeutic substance release and tissue repair [49,50]. Among them, proteins (including, fibroin, collagen, and keratin), polysaccharides (such as, glycosaminoglycans, alginate, chitosan, plant origin natural gum, cellulose, and gellan gum), lipids (such as liposomes and saturated fatty acids), extracellular vesicles (EVs), and extracellular matrix (ECM) have been extensively investigated and are reviewed in detail elsewhere [51,52,53,54,55,56,57,58,59].

## 3. Delivery Strategies for Regenerative Therapy

**Figure 3:**
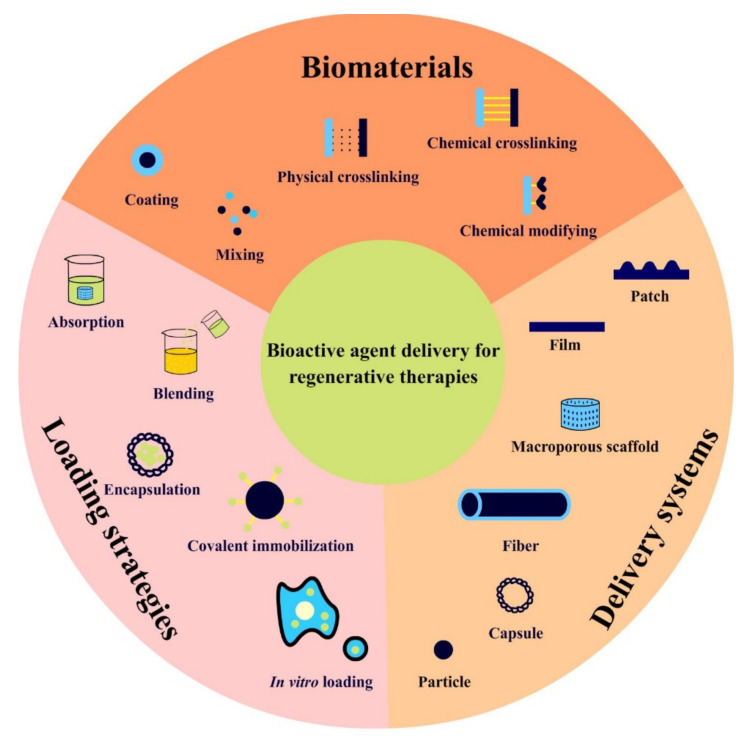
Overview of various delivery strategies for bioactive agent-based regenerative therapies.

To achieve successful tissue regeneration, the design and fabrication of ideal delivery systems are as essential as the characteristics of the bioactive compounds and biomaterials used within them. Specifically, modern delivery systems with high loading efficiency, bioavailability, spatiotemporal control of the release profile, and biological safety are particularly desirable for the treatment of damaged tissues. Various aspects of drug delivery strategies around the key factors (biomaterials, delivery systems, and loading strategies) have been investigated to optimize their physicochemical properties, including biomaterial selection, performance optimization, delivery format, fabrication method, and loading strategies (Figure 3) [60].

### 3.1. Biomaterial Selection and Performance Optimization

Choosing the appropriate biomaterial is critical to the design of a delivery system for regenerative therapy [39,61]. Biomaterial properties that may affect delivery system performance include chemical composition, molecular weight, surface charge drug-carrier interactions, etc. [61,62,63].

Adding small molecules with expected features, combining two or more biomaterials, crosslinking, and chemically integrating functional groups, segments, or molecules are practical methods employed to achieve additional characteristics for biomaterials used as delivery systems (Figure 3) [15,64,65,66,67]. For example, a sonosensitive emulsion was doped in fibrin matrix for the development of an acoustically-responsive delivery system (ARDS) to control angiogenic processes [68]. The release mechanism of this system was acoustic droplet vaporization (ADV), whereby the perfluorocarbon phase within the sonosensitive emulsion vaporized into gas, thus, disrupting droplet morphology, and leading to therapeutic payload release [68]. Implanting four different spatial patterns of ADV within ARDSs in vivo elicited spatially-defined patterns of angiogenesis [69].

### 3.2. Fabrication Strategies for Delivery Systems

A wide range of delivery systems, including micro- and nanoparticles/capsules, hydrogels, fibers, films, patches, and macroporous scaffolds, are currently in use and under development for the sequential delivery of therapeutic substances in synergistic tissue regeneration (Figure 3) [70,71]. Reported fabrication methods for these delivery systems include emulsification, microfluidics, 3D printing, electrospinning, rotary jet spinning, freeze-drying, polymer self-assembly, extrusion, spraying, sol-gel processing, template-assistance, layer-by-layer assembly, solvent evaporation, and cell exosome production [23,35,72,73,74,75,76,77].

In recent years, fabrication strategies for delivery systems have focused on controlling issues that affect drug encapsulation, release, and biological efficacy. In 2021, Gimondi et al. specifically studied the effects of several parameters, including polymer concentration, flow rate, and flow rate ratio, on the size-controlled properties of nanoparticles obtained by microfluidics [23]. The results of this study indicated that flow rate did not lead to significant changes in size distribution, but nanoparticle size increased with polymer concentration [23]. Moreover, 20 nm increments in nanoparticle diameter impacted drug loading, release, and cell response, particularly in an inflammatory environment [23]. Perteghella et al. developed a novel carrier-in-carrier system based on EVs isolated from mesenchymal stem cells incubated with silk/curcumin nanoparticles, which combined the advantages of both pharmaceutical nanomedicine and regenerative cell therapy [27].

More recently, Fahimirad et al. produced electrospun poly(ε-caprolactone)/chitosan/curcumin nanofibers, and then electrosprayed curcumin-loaded chitosan nano-encapsulated particles (CURCSNPs) on their surface [78]. This strategy protected the biological efficacy of the bioactive agents from the adverse processing conditions encountered during electrospinning, resulting in enhanced antioxidant and antibacterial properties and cell proliferation. Furthermore, complete and well-organized wound healing was obtained with CURCSNPs [78].

Strategies to achieve simultaneous or sequential delivery of multiple bioactive agents under anticipated release kinetics are also promising for tissue regeneration applications [11]. As an example, Federico et al. reported the fabrication of an asymmetric double-layer membrane by electrospinning a hydrophobic layer with a ciprofloxacin-loaded polyurethane polycaprolactone copolymer and an ion-responsive hydrophilic layer with a fibroblast growth factor (FGF)-2-loaded octyl derivative of gellan gum/polyvinyl alcohol composite [79]. The results of this study demonstrated that the biological activity and release kinetics of ciprofloxacin and FGF-2 could be controlled by the ionotropic properties of the octyl derivative of gellan gum, thus, realizing controlled delivery according to the needs of different wound healing phases [79]. Alizadehgiashi et al. used 3D bioprinting to develop a cellulose nanocrystal/chitosan methacrylamide composite hydrogel that could be selectively loaded with biologically active ingredients (small molecules, metal nanoparticles, or proteins) [80]. The independent controlled release of multiple bioactive agents enhanced various physiological responses associated with healing, suggesting promise for personalized treatment of various wound types in the future [80]. In addition to its capacity for independent temporal control of bioactive agent delivery, 3D bioprinting can also endow delivery systems with spatial control of delivery by printing hydrogels loaded with different bioactive agents in particular regions of the scaffold to reconstruct complex tissues [81].

### 3.3. Loading Strategies

Various loading strategies (e.g., absorption, adsorption, blending, physical encapsulation, entrapment, covalent immobilization, and in vitro loading) have been utilized to incorporate bioactive substances into delivery systems (Figure 3) [28,65,82,83]. These strategies have been be applied alone or in combination to load one or multiple therapeutic cargos with tunable release kinetics (Table 1). The major factors to consider in choosing a loading strategy include preserving the bioactivity of the therapeutic agent, achieving superior loading efficiency and loading gradients, and realizing spatiotemporal control of bioactive substance release from the delivery system.

To preserve maximum bioactivity, substances should be loaded onto delivery systems under appropriate loading conditions (e.g., solvent, temperature, pH, etc.) according to their physicochemical properties. In addition, blending bioactive agents with stabilizing excipients (e.g., zeolitic imidazolate frameworks, PEG, PVA, sugars, etc.) or prepreparing them as emulsions or microspheres are effective strategies to minimize the adverse impacts of harsh preparation conditions during the fabrication of delivery systems [24,25,78,89,90].

To achieve superior loading capacity, methods that enhance binding affinity, facilitate membrane permeabilization, and optimize delivery system structure have been explored. Modifying the surface of the delivery system with molecules capable of enhancing its binding affinity towards active biomolecules is one feasible strategy [85]. For example, heparin was conjugated on bovine serum albumin-crosslinked PEG aldehyde nanoparticles, which was then used to load vascular endothelial growth factor (VEGF) and bFGF through electrostatic interaction with high efficiency [85]. In addition, molecularly imprinted polymers, synthesized by prepolymerization of functional monomers, also showed good performance in specific recognition and selective adsorption because of their numerous active sites for specific target molecules [86]. Further, therapeutic cargo can be covalently loaded through chemical modification [25,42,43,81,91]. For cell-produced delivery systems, the saponin-assisted method, which facilitates permeabilization, induced up to 11-fold higher loading capacity of a therapeutic substance than passive loading methods [28,92]. Structure optimization strategies mainly include porosity adjustment and microstructure control [26,84]. For example, Radi’c et al. developed nanocoloidal graphene oxide-infused 2-hydroxyethyl methacrylate/gelatin/alginate hydrogels using an adapted porogen leaching method [26]. These delivery systems, which exhibited interconnected porous structures with tunable porosity up to 76%, showed good swelling capacity and high loading efficiency [26]. A continuous supply of sufficient active therapeutic substances is beneficial for tissue regeneration [93]. Therefore, loading strategies that achieve sustained controlled release have become a research focus [61,80,81,88,94]. Recently, goserelin (a water-soluble drug) was loaded on PLGA microspheres using hot-melt extrusion oil-in-water technology, and resulted in reduced initial release but prolonged drug release for nearly 35 days [95]. Bari et al. fabricated lyosecretome-loaded 3D-printed poly(ε-caprolactone)/alginate composites by encapsulating lyosecretome into alginate-based hydrogels, followed by coprinting with poly(ε-caprolactone) [88]. Different from the burst release phenomenon observed from absorption loading-based delivery systems, these lyoscretome-loaded composites showed a promising controlled release behavior that could be altered by changing the composition, spatial structure, and degree of crosslinking of the alginate hydrogel [88]. Other loading methods, such as blending, covalent immobilization, as well as in vitro loading and seeding, can also prevent burst release and achieve long-term sustained release [27,79,80,94]. For instance, silica-coated magnetic nanoparticles can be integrated into cells through endocytosis and may exert targeted delivery function under external magnetic field [96]. Seeding is **a** unique loading method for cells [97]. Salem et al. seeded smooth muscle cells on a triple-layered PLGA sheet for the reconstruction of subtotally resected urinary bladder in rats, resulting in muscular layer regeneration and urothelial ingrowth [98].

## 4. Therapeutic Applications for Lower GU Injuries

### 4.1. Female Reproductive System

#### 4.1.1. Uterus

The uterus, which is composed of the perimetrium, myometrium, and endometrium, is an important female reproductive organ that is essential for embryo implantation and development [99]. Caesarean section, severe infection, and serious injuries may cause the aberrant activation of fibrosis and expression of estrogen receptor alpha, ultimately leading to scar formation and uterine adhesions, which severely affects women’s health [100]. The effects of common treatments, including hysteroscopy adhesiolysis and placement of physical barriers, remain poor in severe cases [101]. Additional therapies for preventing adhesions as well as uterine regeneration and reconstruction involve the administration of therapeutic substances including but not limited to drugs, growth factors, chemokines, and exosomes [20,100,102,103]. However, repeated administration of some therapeutic substances was required due to their poor solubility, high diffusibility, and rapid clearance which resulted in very low drug concentration at the site of injury in the uterus. [104]. As such, suitable therapeutic agent-based delivery systems are urgently in need for the treatment of some uterine disorders or injuries to improve the half-life of the substances, delivery efficacy, and therapeutic effect.

β-estradiol (E2) is an endogenous hormone that has antifibrosis, adhesion prevention, and endometrial regeneration effects [102]. In 2020, a system capable of delivering E2 with a 21-day release profile consistent with the female menstrual cycle was developed by dispersing E2-loaded PLGA microspheres in the amniotic extracellular matrix [105]. In another study, an E2-loaded nanoparticulate decellularized uterus embedded with aloe/poloxamer hydrogel (E2@uECMNPs/AP) was used to treat intrauterine adhesion (IUA) in a rat model [106]. The E2@uECMNPs/AP group exhibited significantly enhanced morphological recovery and less uterine fibrosis than the IUA group, the E2 alone group, the commercially available E2 gel group, and the AP hydrogel group, indicating that this delivery system was capable of effectively promoting endometrial regeneration and preventing re-adhesion [106]. Moreover, considering that sex hormones are key factors to regulate the menstrual cycle phases of endometrium, enzyme expression and hormone metabolism, polymorphisms that exist with these enzymes could also be taken into consideration while designing hormone delivery systems to ensure drug efficacy [107]. Other than estrogen, regenerative therapies delivering bioactive agents such as vitamin C and curcumin have also demonstrated promising results in the treatment of uterine disorders or injuries [101,108].

**Figure 4:**
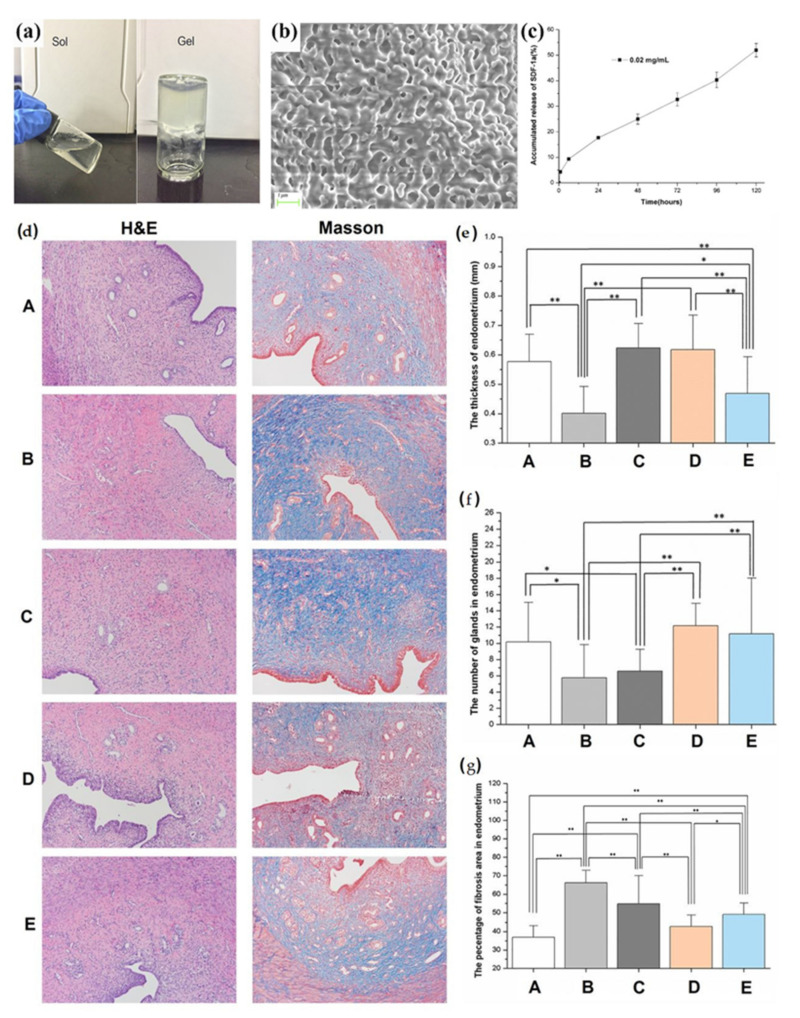
(**a**) Chitosan–heparin solution and chitosan–heparin hydrogel; (**b**) scanning electron microscope image of lyophilized chitosan–heparin delivery system; (**c**) in vitro releasing curves of SDF-1α in chitosan–heparin delivery system; (**d**) hematoxylin and eosin staining and Masson trichrome staining of uterus tissue at 7 days after surgery; The endometrial thickness (**e**), numbers of glands (**f**), and fibrosis area (**g**) of uterus in group A–E at 7 days after surgery. For statistical significance, ** *p* < 0.01 and * *p* < 0.05. A, control group; B, no treatment group; C, hydrogel only treated group; D, SDF-1α hydrogel treated group; E, SDF-1α treated group; SDF-1α, stromal cell-derived factor-1α; H&E, hematoxylin and eosin staining. Adapted with permission from [112], published by Elsevier, 2020.

Several uterine regeneration-related growth factors or chemokines, such as keratinocyte growth factor, bFGF, VEGF, and stromal cell-derived factor-1 (SDF-1) have also been used in delivery-based systems to obtain a controlled spatiotemporal release profile and restore the anatomy and function of the uterus [109,110,111,112,113]. For example, Wenbo et al. designed a chitosan–heparin delivery system for controlling the release of SDF-1α to an injured rat endometrium (Figure 4a–c) [112]. In this study, the number of glands, thickness of the endometrium, and level of fibrosis did not differ between the SDF-1α-treated and the normal control groups after 7 days (Figure 4d–g) [112]. In another study, SDF-1α carried by a silk fibroin-bacterial cellulose membrane was employed to treat full-thickness uterine injury [100]. This SDF-1α-loaded delivery system showed promising effects on arteriogenesis, mature endometrium formation, and pregnancy outcome [100]. Further, Jiang et al. successfully constructed a collagen-targeting bFGF delivery system by fusing a collagen-binding domain peptide to the N-terminal of bFGF [114]. Administration of this bFGF delivery system around the scarred endometrium of 18 uterine-infertile women every 4 weeks improved endometrial thickness, scarring of the endometrial area, vascular density, and menstrual blood volume [103]. Notably, three of the 18 bFGF-treated patients achieved pregnancy over 20 gestational weeks [103]. Liu et al. loaded stem cell secretome, which contained a great variety of regeneration-related growth factors, on a crosslinked hyaluronic acid hydrogel to facilitate the sustained release of growth factors and support the morphological and functional recovery of the uterus [115]. This approach demonstrated great potential for clinical translation because of its long shelf-life and superior safety profile.

#### 4.1.2. Ovaries

The ovaries, which are composed of the outer layer, capsule, cortex, and medulla, are paired organs that produce oocytes and reproductive hormones [107]. Age, disease, chemotherapy, or radiotherapy may cause various ovarian diseases, such as primary ovarian insufficiency, ovariectomy, and polycystic ovary syndrome [116]. These diseases can result in hormonal imbalances, metabolic syndrome, infertility, and genital atrophy, which threatens the health of women [99,117]. Current treatments generally include hormone replacement therapy and ovary transplantation. Growth factors that dominate follicular survival, latency, activation, and maturation, such as growth and differentiation factor-9 (GDF-9), bone morphogenetic protein-4 (BMP-4), VEGF, platelet derived growth factor (PDGF-ββ), and anti-mullerian hormone, are promising bioactive agents for delivery-based regenerative therapy to restore ovarian function [118,119,120]. For example, VEGF-loaded fibrin-heparin-binding peptide hydrogel was engineered to promote ovarian graft survival in a bilateral ovariectomy mouse model [119]. This delivery system prolonged VEGF activity and release, thereby, promoting angiogenesis, enhancing engraftment, and improving the function of the transplanted ovarian tissue [119]. Additionally, growth factors can be combined for better therapeutic effect. For example, a macroporous alginate scaffold loaded with BMP-4, GDF-9, VEGF, and PDGF-ββ was shown to restore the ovarian function of ovariectomized mice [120]. This regenerative system helped support the follicle reach to antral size, retained hormone-secreting function, and resumed cyclic vaginal appearance, thus, leading to the restoration of ovarian function in vivo [120].

In addition, some living cells, as autologous constant sources of active biomolecules, have been used in delivery-based systems to treat ovarian dysfunction [121,122,123,124]. Green et al. designed a delivery system encapsulating adipose-derived stem cells (ADSCs) in crosslinked alginate beads [125]. The alginate/ADSCs group displayed higher follicle survival and better follicle growth, antrum formation, and oocyte maturation than the follicles cultured alone group, suggesting that cytokine excretion by ADSCs may play an important role in the maturation of early-stage follicles [125]. In 2021, a layer-by-layer form of follicle spheroids composed of autologous ovary cells, gelatin, and/or Matrigel was applied in the treatment of ovarian endocrine function loss [123]. This system significantly restored the endocrine function of ovariectomized rats and induced fewer side effects than in rats treated with synthetic hormones [123]. In another study, bone marrow-derived mesenchymal stem cells (BMSCs) were used to deliver the regulatory factors necessary for estrogen production [124]. The BMSCs-containing system effectively promoted stable and long-term estrogen secretion, regulated pituitary hormones, and improved physiological outcomes in an ovariectomized rat model [124].

#### 4.1.3. Cervix and Vagina

The cervix, which is composed of ectocervix, the cervical transformation zone, and the endocervix, plays an important role in uterine growth and fetal development, the dysfunction and abnormality of which may cause premature birth [126]. Cervix cancer is a class of common cervix diseases usually treated by transabdominal hysterectomy. Regenerative therapies may provide promising strategies to reconstruct the structure and preserve the reproductive and physiological function of this kind of cervical defects. Zhao et al. engineered an 3D-printed cervix-like implant with drug release function, which was loaded with anti-human papillomavirus protein under negative pressure [127]. Validation of the quantitative loading and release capacity of this engineered delivery system, which inhibited dissociated virus near the cervix, suggested a promising functional tissue implant for patients whose cervixes have been resected due to human papilloma virus -induced cancer [127].

The vagina is an elastic muscular tube composed of vaginal mucosa, an intermediate muscle layer, and the adventitia. Among them, the vaginal mucosa, whose glycogen content can be adjusted depending on estrogen levels and undergoes cyclical changes, serves as a barrier to the entry of pathogens [128]. Its barrier function is enhanced by the acidic microenvironment created by *Lactobacilli* colonized in the vagina [129]. In this regard, to avoid potential dysbiosis, the vaginal microbiome should be taken into account when designing delivery systems [107]. Vagina regenerative medicine focuses on the treatment of acquired vaginal trauma, deformity, and agenesis caused by trauma, surgical operation, or disease at birth. In 2021, umbilical cord mesenchymal stem cell-loaded small intestinal submucosa was bioengineered to reconstruct a damaged vagina in a rhesus monkey [130]. The in vitro study demonstrated that this regenerative system produced several bioactive substances, such as elastin, hepatocyte growth factor, insulin-like growth factor, and VEGF [130]. Three months after transplantation, the engineered vagina showed enhanced ECM reorganization, large muscle bundle formation, angiogenesis, and mechanical properties, thus, suggesting a new approach for the repair of vaginal injury [130].

### 4.2. Male Reproductive System

Dysfunction in the male reproductive system is the main cause of male infertility, which heavily affects male reproductive health [131,132,133,134]. Fortunately, regenerative therapy offers an opportunity for these patients to recover and even become biological fathers. Recently, an alginate matrix containing necrosis inhibitor nanoparticles (NECINH-NPS) was used to encapsulate testicular tissue fragments and achieve better reproductive outcomes [135]. After orthotopic auto transplantation, germ cell survival and testicular tissue integrity were found to be significantly improved [135]. Further, calcium-alginate hydrogels encapsulated with VEGF-nanoparticles, PDGF-nanoparticles, and NECINH-NPS have been used to optimize the angiogenesis of transplanted testicular tissue [136]. An in vivo study confirmed that the combined delivery of VEGF and PDGF nanoparticles effectively promoted vascular maturity [126]. In another study, curcumin-loaded iron oxide particles (CIOP) were utilized to treat scrotal hyperthermia-induced azoospermia [134]. The CIOP-treated group exhibited increased testes volumes and seminiferous tubule lengths, as well as improved sperm parameters, stereological parameters, and serum testosterone levels as compared with the scrotal hyperthermia group [134]. Ghorbani et al. incorporated multi-walled carbon nanotubes into poly(L-lactic acid) fibers to deliver naringenin for the regeneration of impaired spermatogenesis [137]. The beneficial role of naringenin, an effective antioxidant, in spermatogenesis was demonstrated in vitro by measuring the reduction in ROS generation in spermatogonial stem cells treated with different concentrations of naringenin [137].

In addition to fertility restoration, regenerative therapy has effectively treated other andrology-related diseases, such as erectile dysfunction and testosterone deficiency [131,133]. For example, ADSCs incubated with NanoShuttle (biocompatible magnetic nanoparticles) were employed to improve cavernous nerve injury-induced erectile dysfunction in a rat model [138]. ADSCs survival was improved under the protection of NanoShuttle and cells remained in the corpus cavernosum for at least 3 days [138]. Additionally, the smooth muscle, endothelium, and nerve tissue were significantly improved in the NanoShuttle/ADSCs group as compared with those in the cavernous nerve injury group without treatment, resulting in favorable erectile dysfunction treatment effects [138].

### 4.3. Urinary System

Regenerative therapy is necessary following various irreversible physiological disorders of the urinary system, including hypospadias, urethral stricture, end-stage bladder disease, and urinary incontinence [128,139].

To increase therapeutic efficacy, growth factors (e.g., insulin-like growth factor 1 (IGF-1), transforming growth factor beta-1 (TGF-β), VEGF, etc.) have been loaded on various delivery-based systems to obtain desired properties [140,141,142]. Yan et al. demonstrated the superiority of IGF-1-loaded alginate-poly-L-ornithine-gelatin (A-PLO-G) microbeads over empty A-PLO-G microbeads to regenerate an external urethral sphincter defect in a stress urinary incontinence (SUI) rat model [142]. The IGF-1-loaded A-PLO-G group showed enhanced regeneration effects, such as well-organized skeletal muscle fibers and vascular development as compared with the group treated with saline or empty A-PLO-G microbeads [142]. Based on these findings, the researchers suggested that promoting skeletal myogenesis and revascularization by periurethral administration of IGF-1-loaded A-PLO-G microbeads may facilitate recovery from SUI [142]. Moreover, Ardeshirylajimi et al. studied the efficacy of a poly (vinylidene fluoride) (PVDF) electrospun fiber scaffold loaded with TGF-β/chitosan nanoparticles for the functional recovery of reconstructed bladders [141]. This delivery system exhibited a long-term sustained release profile, and the bioactive agent-treated group exhibited significantly increased ADSCs proliferation and smooth muscle cells differentiation as compared with groups treated with PVDF and tissue culture polystyrene, suggesting promise for ladder tissue engineering applications [141].

**Figure 5:**
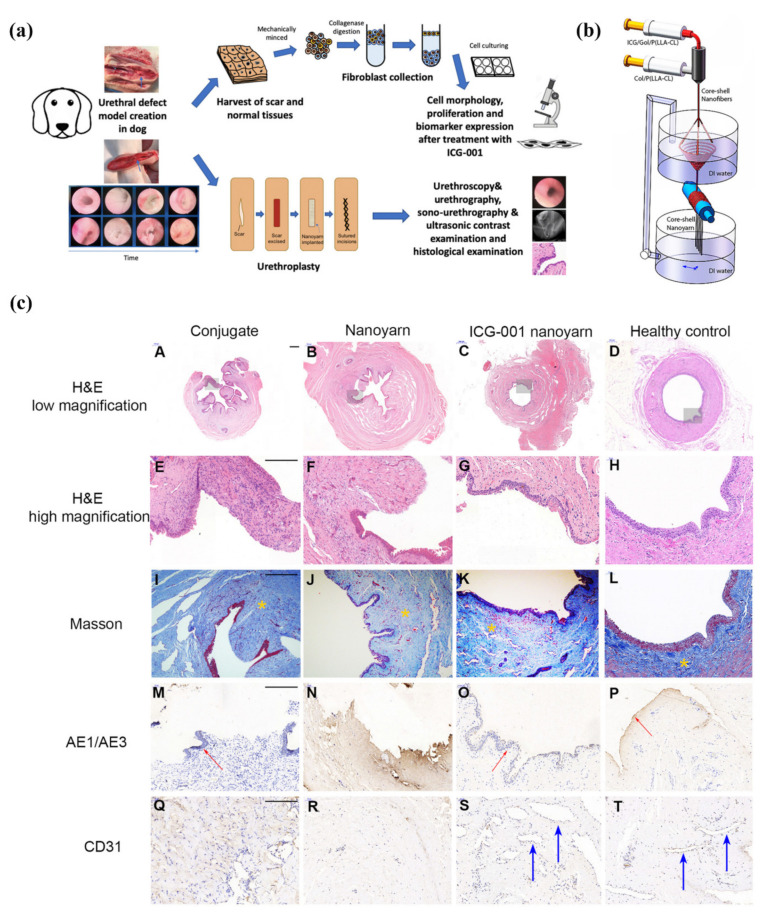
(**a**) Study design of using ICG-001 loaded collagen/poly(L-lactideco-caprolactone) nanoyarn to treat urethral defects in a canine model; (**b**) fabrication process of ICG-001 delivered collagen/poly(L-lactideco-caprolactone) nanoyarn; (**c**) hematoxylin and eosin (A–H), Masson trichrome (I–L), AE1/AE3 (M–P) and CD31 (Q–T) staining of urethras in each group at 12 weeks post urethroplasty. H&E, hematoxylin and eosin. Red arrows indicate the epithelium in (M,O,P). Blue arrows indicate vessels in (S,T). Yellow asterisks indicate ECM in (I–L). Adapted with permission from [143], published by Frontiers, 2020.

Further, a canonical downstream signaling pathway of TGF-β, Wnt/β-catenin, plays a crucial role in the tissue repair process. For example, ICG-001, a well-established Wnt signaling inhibitor, was loaded on a collagen/poly(L-lactideco-caprolactone) nanoyarn with a core-shell structure to treat urethral defects in a canine model (Figure 5a,b) [143]. This ICG-001-delivery system induced decreased fibroblast proliferation, suppressed fibrotic protein expression, and restored a fully functional urethra within 12 weeks (Figure 5c) [143].

Cell-based delivery systems are another promising approach for urinary tissue regeneration. In one clinical study, autologous buccal epithelial cells encapsulated in thermos-responsive hydrogel were implanted at the stricture site of six male patients with bulbar urethral stricture [144]. All patients recovered with healthy mucosa at the urethrotomy site and voided wells [144]. Although two patients reported recurrence within two years, this delivery system may be beneficial for the treatment of urethral stricture [144]. Wang et al. developed a bladder patch capable of localized non-invasive delivery of transplanted cells in vivo by utilizing ADSCs-labeled ultrasmall SPIONs and porous polyglycolic acid scaffolds [145]. This delivery system not only promoted the regeneration of bladder tissues (urothelium, smooth muscle, neural cells, and blood vessels) but also restored bladder function with augmented capacity, which holds great therapeutic promise for bladder injuries [145]. Furthermore, cell- or tissue-derived EVs and ECM, which contain multiple active biomolecules, can confer desirable biochemical properties to delivery systems, thereby, achieving urinary regeneration effects [21,146,147].

## 5. Conclusions and Future Perspectives

Tireless efforts have been made to develop spatiotemporal controlled release systems to deliver bioactive agents with optimal activity, stability, and safety to enhance their curative effect. In general, biologicals released under a controlled behavior in spatial dimension can be obtained by building preferred anisotropic constructs, while controlled behavior in the temporal dimension can be achieved by biomaterial modification and active biomolecule system interactions. The interplay between the consideration of the biomaterials, the delivery systems, and the drug-loading strategy plays a pivotal role in the generation of new delivery systems for regenerative products. Significant advances in biomaterial design and modification, delivery systems fabrication, and drug-loading methods have allowed the development of better and more diverse functionalities, and more precise and effective bioactive agent delivery systems in recent years. Current research describes delivery systems loaded with therapeutic substances for the treatment of lower GU injuries, which have resulted in improved effectiveness through direct administration.

Although marvelous progress has been made, there are still many aspects of delivery-based regenerative therapy that need further exploration. The challenges in clinical translation and potential ethical problems related to transplantable reproductive tissues still need to be addressed. Considering that the biological requirements of different cells in damaged tissues at different regeneration stages are varied, future studies should focus on the multimodal combination of active biomolecules with tailored release behavior, thus, resulting in a regenerative system that can provide the adequate sequential delivery needed for tissue formation and better therapeutic effect. To facilitate this, 3D bioprinting is a promising delivery approach to develop regenerative products with patient-specific, tailored properties. Regenerative scaffolds, especially those involving novel biomaterials and complex delivery systems, may increase potential security risks that will be detrimental to clinical translation. It might be necessary to establish a complete workflow regarding the novel delivery system-based regenerative scaffolds from delivery system manufacturing practice standards to comprehensive preclinical and clinical studies to achieve translation to the clinic.

It is worth noting that under the rigorous efforts from engineers, biologists, clinicians, and other related disciplines, the ongoing development is expected to create innovative, safe, and effective delivery systems that hold great promise for the clinical treatment of lower GU injuries in the coming decades.

## Figures and Tables

**Table 1:** List of multiple loading strategies utilized in bioactive agent-based delivery systems for regenerative therapy with varied release kinetics.

Loading Strategy.	Bioactive Agent	Delivery System	Release Kinetics	Release Time (Days)	Ref.
Absorption	FGF-2	Hydrogel	Sustained release	3	[84]
Absorption	Heparin	Hydrogel	Sustained release	14	[19]
Absorption	Curcumin	Hydrogel	Burst then sustained	1.25	[26]
Adsorption	bFGF and VEGF	Nanoparticle in electrospun nanofiber	Burst then sustained	14	[85]
Adsorption	4-Aminopyridine	Electrospun nanofiber	Burst then sustained	4	[86]
Blending	Curcumin	Electrospun nanofiber	Controlled release	3.5	[87]
Blending	Ciprofloxacin and FGF-2	Electrospun membrane	Sustained release	7	[79]
Blending	AgNPs, VEGF, BSA, and gentamycin	3D-printed hydrogel	Burst then sustained	3	[80]
Blending	Secretome	3D-printed macroporous scaffolds	Sustained release	10	[88]
Immobilization	CTGF and TGF-β3	Nanoparticle in 3D-printed scaffold	Controlled release	35	[81]
Immobilization	bFGF	Nanoparticle	Sustained release	12	[25]
Immobilization	AT-RvD1, IL-10	Hydrogel	Sustained release	5	[42]
Immobilization	bFGF	Hydrogel	Controlled release	8	[43]
Encapsulation	hGDF-5	Nano-carrier in hydrogel	Controlled release	18	[41]
Encapsulation	bFGF	Emulsion in hydrogel	Ultrasound-controlled release	8	[68]
Encapsulation	Curcumin	Nanoparticle in nanofiber	Sustained release	16	[78]
Encapsulation	Curcumin	Hydrogel	Controlled release	0.25	[61]
Entrapment	Curcumin	Nanoparticle in extracellular vesicle	Burst then sustained	4	[27]

FGF-2, fibroblast growth factor-2; bFGF, basic fibroblast growth factor; VEGF, vascular endothelial growth factor AgNPs, silver nanoparticles; BSA, bovine serum albumin; 3D, three-dimensional; CTGF, connective tissue growth factor; TGF-β3, transforming growth factor-β3; AT-RvD1, aspirin-triggered resolvin D1; IL-10, recombinant human interleukin 10; hGDF-5, human growth differentiation factor 5.
